# Systemic Inflammation after Aneurysmal Subarachnoid Hemorrhage

**DOI:** 10.3390/ijms241310943

**Published:** 2023-06-30

**Authors:** Chang-Zhang Chai, Ue-Cheung Ho, Lu-Ting Kuo

**Affiliations:** 1 Department of Medical Education, National Taiwan University, School of Medicine, Taipei 100, Taiwan; changzhangchai@gmail.com; 2 Division of Neurosurgery, Department of Surgery, National Taiwan University Hospital Yunlin Branch, Yunlin 640, Taiwan; coolive0510@hotmail.com; 3 Division of Neurosurgery, Department of Surgery, National Taiwan University Hospital, Taipei 100, Taiwan

**Keywords:** cerebral aneurysm, subarachnoid hemorrhage, systemic inflammation

## Abstract

Aneurysmal subarachnoid hemorrhage (aSAH) is one of the most severe neurological disorders, with a high mortality rate and severe disabling functional sequelae. Systemic inflammation following hemorrhagic stroke may play an important role in mediating intracranial and extracranial tissue damage. Previous studies showed that various systemic inflammatory biomarkers might be useful in predicting clinical outcomes. Anti-inflammatory treatment might be a promising therapeutic approach for improving the prognosis of patients with aSAH. This review summarizes the complicated interactions between the nervous system and the immune system.

## 1. Introduction

Subarachnoid hemorrhage (SAH) represents 3% of all of causes of stroke [1], with a crude incidence rate of 7.9 per 100,000 person years globally [2]. Aneurysmal SAH (aSAH) accounts for approximately 85% of spontaneous SAH events [3], while other causes include trauma, arteriovenous malformation, infection, tumor, etc. Depending on the size and position of the hematoma, patients with SAH report severe headaches, neck stiffness, elevated blood pressure, loss of consciousness, and neurological deficits [4]. Cranial computed tomography (CT) and magnetic resonance imaging (MRI) are useful tools for the diagnosis of SAH on the day of incidence; however, MRI is superior weeks after the hematoma has subsided [5]. SAH is one of the deadliest brain injuries, with a high early mortality rate and severe long-term neurological sequelae [6]. Factors influencing prognosis include the pre-aSAH status (i.e., medical history of hypertension, previous SAH event, and old age), SAH severity, and post-aSAH complications [7]. Specifically, the inflammatory response following aSAH may mediate various post-aSAH complications and impact clinical outcomes.

This review summarizes all the aspects of the systemic inflammatory responses following aSAH, focusing on the predictive value of the biomarkers for outcome and providing an update on currently ongoing clinical trials tackling systemic inflammation following aSAH. A literature search was conducted in the Medline/PubMed database, using the keywords “aneurysmal subarachnoid hemorrhage with systemic inflammation”, “systemic inflammatory response syndrome (SIRS)”, “fever”, “biomarker”, “acute phase protein”, “cytokines”, “cerebral vasospasm”, “delayed cerebral ischemia”, “hydrocephalus”, and “anti-inflammatory treatment.” Duplicated and irrelevant articles were excluded after reviewing the abstracts of the identified articles. Similar keywords were used in the clinical trials registry of the US National Institute of Health (www.clinicaltrials.gov, accessed on 30 November 2022) to retrieve records of clinical trials without available published results.

## 2. Neuroimmune Axes in aSAH

The nervous system plays an essential role in mediating immune reactions in trauma patients [8,9,10,11]. This nervous–immune system connection involves both the sympathetic and parasympathetic nervous systems. Patients with SAH experience a tremendous amount of physiological stress during rupture of an aneurysm, which activates the sympathoadrenal pathway and induces a catecholamine surge [12,13,14]. The catecholamine surge was suggested to promote the production of chemokines and cytokines via the hypothalamus–pituitary–adrenal (HPA) axis [15,16], resulting in systemic inflammatory responses [17]. This can cause cardiac (i.e., neurogenic stress cardiomyopathy), pulmonary (i.e., lung parenchymal inflammation), and renal (i.e., acute kidney injury) complications, as well as cytokine release syndrome (CRS) [18,19].

It was reported that 15.8–41% of CNS injury patients contracted nosocomial infections during hospitalization [20,21,22,23]. Increased infection risk (31% vs. 17%) was observed among patients with non-traumatic SAH, with lymphopenia reported among 45% of the patients [24]. The inflammatory cytokine surge may contribute to the immunosuppressed state [25,26] after aSAH by promoting the secretion of corticotropin-releasing hormone (CRH) from the hypothalamic paraventricular nucleus (PVN) [27,28,29,30]. This promotes production of ACTH and glucocorticoids, which may explain the reduced NK cell and T lymphocyte levels within the week following aSAH [31].

Through the cholinergic anti-inflammatory pathway, the vagus nerve activates the α-7-nicotinic acetylcholine receptor (α7nAChR) on macrophages, reducing tumor necrosis factor-α (TNF-α) secretion [32,33]. The same pathway was found to be responsible for the inhibition of inflammatory cells in the spleen [34,35,36]. Results from a rodent SAH model indicate that splenectomy helps to reduce systemic inflammation and prevents tissue injuries in cardiac and nervous systems [37]. Peripheral interleukin (IL)-1β may also activate the afferent vagus nerve and promote CRH production, further suppressing the immune system [38].

The nervous and immune systems also communicate through the glymphatic system, which is the essential pathway for eliminating metabolic waste from the brain to sustain homeostasis [39]. Erythrocyte- and brain-specific antigens were found in the deep cervical lymph node following ischemic stroke and SAH, confirming the lymphatic-system–brain connection [40,41]. In aSAH rodent model studies, glymphatic channel disruption was found to promote perivascular neuroinflammation due to accumulation of heme waste products in the subarachnoid space [42,43]. Disruption of the blood–brain barrier and glymphatic system in stroke may facilitate the exposure of brain-specific antigens to the adaptive immune system, in turn inducing autoimmune disorders, resulting in chronic inflammation and, potentially, dementia [44].

## 3. Tissue Injury Mechanism in aSAH: Primary, Secondary, and Extracranial

aSAH tissue injuries can be divided into primary, secondary, and extracranial injuries according to the timing and mechanism. The fundamental mechanism of primary tissue damage in aSAH is attributed to the mechanical pressure from the subarachnoid hematoma [45]. In contrast, global cerebral ischemia and toxic hemoglobin metabolites lead to secondary injuries of aSAH via cytotoxic, oxidative, and inflammatory pathways [46,47,48,49,50,51]. Studies suggest that both neuroinflammation [52] and systemic inflammation [53] are involved in this inflammatory response. The local inflammatory event is marked by an increase in leukocytes and microglia in the subarachnoid space and brain parenchyma [54], while systemic inflammation can be easily detected using readily available clinical tools (i.e., body temperature, routine blood test, inflammation index, etc.). Upregulated systemic proinflammatory mediators may also, in turn, promote local inflammation [55,56,57,58,59,60]. Integrating inflammation-suppressive treatment into aSAH care is gaining popularity, with more evidence suggesting the role of inflammation in secondary aSAH injuries [61,62].

The secondary injuries in aSAH can be further categorized into early brain injury [63,64], cerebral vasospasm [65], delayed cerebral ischemia (DCI) [66], and chronic hydrocephalus [67]. Early brain injury describes clinical symptoms with progressing brain edema, develops within 72 h after SAH, and commonly occurs among patients with worse initial disease grading (Hunt and Hess scale > 4 or World Federation of Neurosurgical Societies (WFNS) grading > 4) [63]. Vasospasm is referred to as the narrowing of the cerebral artery following aSAH, which may result in compromised cerebral blood supply. Vasospasm can be detected by increased flow velocity in the cerebral artery (middle or anterior, >120 cm/s under transcranial Doppler ultrasonography) [65,68] or reduced vessel diameter (>50% under angiography) [69]. DCI is the occurrence of a new neurological defect or suddenly deteriorated consciousness (Glasgow Coma Scale reduced by 2 points) that persists for more than 1 h without other explanation [65] and usually occurs 4 to 14 days after aSAH [70].

Extracranial complications include fever, hemodynamic dysregulations, anemia, electrolyte imbalance, cardiac dysfunction [37,71], pulmonary injury [72], coagulation cascade activation, and nosocomial infections [73]. Possible mechanisms of extracranial complications include catecholamine hyperactivity, hemodynamic dysfunction, and systemic inflammation. Kurtz et al. found that the severity of non-neurological systemic organ dysfunction, as measured by nSOFA score, was related to aSAH hospitalization mortality [74]. Neurogenic pulmonary edema is associated with poor clinical outcome and believed to be closely associated with systemic inflammatory response [72]. Elevated inflammatory cytokines may increase pulmonary capillary permeability, disrupt cardiovascular functions, and promote neutrophil migration into pulmonary parenchyma. The pulmonary tissues are thus damaged under the effect of increase production of reactive oxygen species (ROS) and elastase [72,75,76]. Han et al. suggested that the administration of dexmedetomidine significantly improved the neurological outcome and reduced acute lung injury via an anti-inflammatory effect (lower serum IL-6, *p* < 0.05) in a mouse SAH model. [77]. Interferon-β also effectively reduced pulmonary inflammation (i.e., neutrophilic infiltration and TNF-α mRNA expression) in an SAH mouse model, which may improve clinical outcome [78].

## 4. The Initiation of Inflammatory Cascade in aSAH

The inflammatory cascade following aSAH starts with the interaction between DAMP molecules (i.e., hemoglobin metabolites and neuronal damage signals) and local cellular components (microglia, neurons, and endothelial cells) [79]. Intracellular molecules (e.g., HMGB-1 and S100B) are released into the extracellular space as a result of nervous tissue injury. The phagocytic cells and astrocytes pick up these signals through pattern recognition receptors (PRRs) [80], TLR-4, and receptor for advanced glycation end products (RAGE) [81,82,83]. This upregulates the transcription factor, nuclear factor-κB (NF-κB), and boosts the production of inflammatory cytokines (e.g., TNF-α and IL-1β [84]) and chemokines [80,85], subsequently promoting a systemic inflammatory reaction.

Following the accumulation of arterial blood in the subarachnoid space, the erythrocytes are hemolyzed and degraded [46]. Hemoglobin, which consists of a tetramer of four globin chains and a heme group in the erythrocyte, degrades into methemoglobin, heme, and hemin. These metabolites act as DAMPs to activate microglia via the TLR4-dependent pathway and endothelial cells via the TLR4-independent pathway [86,87,88,89,90]. The activated TLR4/MyD88 pathway upregulates NF-κB, the common transcription factor of proinflammatory mediators (e.g., TNF-α, IL-1, and IL-6) [91,92]. Activated microglia release proinflammatory cytokines and boost neutrophil and macrophage migration [53,93], speeding up waste removal. Evidence also shows that heme induces the formation of neutrophil extracellular traps, which may promote thrombosis [94]. These metabolites promote neuroinflammation, endothelial cell apoptosis, and glial cell necrosis, which may damage nervous tissue [95,96]. Heme iron can undergo a Fenton reaction and release ROS, activating the eicosanoid pathway [97,98,99], which may result in a tissue-damaging self-perpetuating loop [99,100].

High-mobility group box 1 (HMGB-1) is a transcription factor found in most eukaryotic nuclei [101] that maintains nucleosome architecture and aids in transcription [102,103]. After aSAH, increased production of HMGB-1 was found in necrotic brain parenchyma [101], specifically from neurons (<1 day) [80] and microglia (>2 days) [104]. Patients with aSAH with high serum HMGB-1 levels have an increased risk of cerebral vasospasm, higher in-hospital mortality, and worse functional outcome at one year [85]. Hemmer et al. found that high serum HMGB-1 levels at admission were associated with an inflammatory state (i.e., higher C-reactive protein (CRP) level and peripheral white blood cell counts) and the risk of DCI in a study of 83 patients with aSAH [105]. In a recent endovascular puncture SAH rat model, Guo et al. suggested that HMGB1 inhibition could reduce neuroinflammation, preventing blood–brain barrier (BBB) disruption and brain edema [106].

S100B is a calcium-binding protein found in glial cells (i.e., astrocytes, microglia, and oligodendrocytes) and regulates cytoskeleton activity, survival signaling, and calcium balance [107]. S100B is released into interstitial space following glial cell death [108]. Elevated S100B levels in peripheral blood indicate blood–brain barrier disruption [109], which is commonly detected within 3 days after aSAH [110]. In a prospective single-center trial of 56 patients with aSAH, Cenik et al. found that S100B was associated with clinical severity [111] and patient outcome [112] after aSAH. Kedziora et al. discovered similar findings and proposed the peripheral S100B level as a good biomarker for mortality prediction (area under the curve (AUC) = 0.825) [113]. S100B may be a useful biomarker to detect post-aSAH infarction and secondary injuries. Uryga et al. found that day-3 S100B blood levels predicted DCI, with an AUC of 0.77 [110], but was not predictive of cerebral vasospasm [110,112]. In the non-DCI group, the first-day serum S100B level was associated with CRP.

## 5. SIRS in aSAH

SIRS is defined as a systemic inflammatory event with two or more of the following conditions: altered body temperature (>38 °C or <36 °C), leukocytosis or leukopenia (>12,000 cells/μL or <4000 cells/μL), tachypnea (>20 breaths/min), and elevated heart rate (>90 bpm) [114]. It is widely used to spot inflammation in infectious and non-infectious events, including autoinflammatory disease and trauma [115,116]. SIRS occurs in 30% to 86% of patients with aSAH [56,59,60,117] and is thought to be a useful marker to predict secondary brain injuries and long-term clinical outcomes [56,60,118]. Furthermore, SIRS criteria (i.e., increased white blood cell count and body temperature) were associated with worse prognosis among patients with aSAH [119]. In a retrospective study of 997 patients with SAH, Kurtz et al. found that many non-survivors significantly experienced SIRS during hospitalization (42% of survivors vs. 63% of non-survivors, *p* < 0.001) [74].

Potential mechanism of induction of SIRS include overspilling of inflammatory cytokines from the intrathecal space produced by activated microglia [120,121] and a catecholamine spike following aSAH [56]. Risk factors for developing SIRS include worse clinical status at initial presentation (World Federation of Neurological Surgeons (WFNS) grade < IV) [56,59,60], intraventricular hemorrhage (IVH) [56], intracerebral hemorrhage (ICH) [122], and aneurysm clipping treatment [56].

SIRS can lead to secondary multiple organ failure syndrome, a leading non-neurogenic cause of death in aSAH [56,60]. Systemic inflammation may impair local tissue perfusion, cause microthrombus formation, and damage organs [53,123,124]. Proinflammatory cytokines (i.e., TNF-α, IL-1, IL-6, etc.) in systemic circulation may block bone marrow erythropoiesis, worsen brain oxygen supply, and induce ischemia [125]. Targeting SIRS to enhance clinical outcomes had therefore become an increasingly popular treatment approach [56,60].

The SIRS score, derived from the number of SIRS criteria met in terms of inflammatory activity [122], has been used to predict outcomes in septic and trauma patients [126,127]. Hokari et al. found that patients with aSAH with a higher SIRS score on day 3 of aSAH had worse functional outcome (modified Rankin Scale (mRS) ≥ 3) upon 3-month follow-up [122]. In a trial of 243 patients with aSAH, Sampson et al. found that a higher initial SIRS score (SIRS score ≥ 3) was associated with higher odds (OR = 5.65) of developing anemia (hematocrit < 30%) within 2 weeks, which is associated with post aSAH brain infarction and worse patient outcome [125].

## 6. Hyperthermia following aSAH

Evidence shows that the overactive inflammatory responses in aSAH are correlated with worse patient outcomes. However, it might be appropriate to consider the inflammatory response in aSAH to be a double-edged sword when taking account of the possible benefits of inflammation in cell debris clearance and tissue regeneration [128,129]. Biomarkers and inflammatory cells in CSF, brain parenchyma, and cerebral vessels may represent intracerebral inflammation, while peripheral blood biomarkers may indicate a global inflammatory state [67,130]. A good biomarker to track systemic inflammation [53,56,131] is necessary for optimum clinical care and improved clinical outcomes. Due to their unique role in systemic inflammation, fluctuations of proinflammatory cytokines, acute phase proteins, and body temperature may each reflect different stages [132]. Fever, or hyperthermia, is a common sign of systemic inflammation. Approximately 23–70% of patients with aSAH experience fever during hospitalization [133,134,135,136,137,138,139]. The criteria of fever were inconsistent in earlier studies, with a body temperature higher than 38.0 to 38.3 °C used as a threshold in different studies [135,140,141]. Among febrile patients with aSAH, less than 50% had a proven infection [135,141], which led to unnecessary antibacterial therapy [133].

A neurogenic fever should be considered after other differential diagnoses have been ruled out [142]. An early-onset fever (within 72 h [133]) with a prolonged episode would also support confidence in diagnosis. Fever was found to be associated with secondary tissue injuries in aSAH, notably vasospasm [140,143] and DCI [141], which suggest the need for intensive treatment [140]. In a retrospective study of 175 patients with aSAH, Saripalli et al. found that DCI was related to a fourth-day body temperature elevation greater than 2.5 °C above baseline (OR: 4.55; 95% CI: 1.31–15.77; *p* = 0.017). Fever reduction has been proposed to enhance clinical outcomes [144].

Subarachnoid and intraventricular hemorrhages were more prone to cause central fever among neurologic intensive care unit (NICU) patients [133]. Leukocytosis, older age, and worse early clinical presentation also increased the neurogenic fever risk [137,145]. The underlying mechanism of non-infectious fever may include direct and indirect hypothalamic disruption [146] and increased proinflammatory cytokine expression in systemic circulation and the intracranial compartment [147]. Circulating proinflammatory mediators increase endothelial cell and perivascular macrophage prostaglandin E2 (PGE2) expression [148]. Peripheral PGE2 from the liver and lung macrophages can also cross the intact BBB via a specialized transporter [149]. The preoptic hypothalamic region detects prostaglandin signals [150,151] and raises the body temperature homeostatic set point [152].

As PGE2 pathway inhibition studies have failed to produce satisfactory clinical improvement, there is increasing interest in exploring new thermogenesis mechanisms in post-aSAH fever [153]. Thomas et al. proposed a new explanation for post-aSAH fever [154]. They defined SAH-induced pyrexia (SAHiP) as a body temperature above 38.5 °C without an infectious source during the week of admission. They found that the fever burden was unchanged in a prostaglandin EP3 receptor knockout or meloxicam-treated mouse SAH model, while global TLR4 knockout mice had a lower core body temperature. Thomas et al. proposed that microglia TLR4 is the key effector in SAHiP development because knockout mice had no fever when microglia TLR4 was eliminated, regardless of peripheral macrophage and neutrophil expression. Tupone et al. examined the role of COX/PGE2 and proposed that brown adipose tissue (BAT) may contribute to neurogenic fever [155]. The results from animal models suggested that the degraded erythrocyte in the subarachnoid space triggered brown adipose tissue, thus raising body temperature. The study also demonstrated that the BAT thermogenesis could be reduced systemically via the administration of N6-cyclohexyladenosine, a strong adenosine A1 receptor agonist.

## 7. Inflammatory Cytokine in aSAH

After SAH, various cytokines (i.e., IL-1, IL-6, TNF-α, etc.) have been found to be increased in both plasma and cerebral spinal fluid (CSF) [156,157] and reported to be associated with clinical outcomes. In a prospective study of 43 patients with aSAH, Al-Tamimi et al. found that CSF proinflammatory cytokine levels (i.e., IL-1b, IL-4, IL-6, IL-8, IL-10, IL-15, Il-17, and TNF-α) were much higher than peripheral blood levels [158]. Based on the concentration gradient between the two compartments, it was proposed that overspilling from the intrathecal area through the disrupted BBB may cause peripheral blood proinflammatory cytokine upregulation [159,160]. Although cytokines were upregulated in both peripheral blood and cerebral spinal fluid [161], Gruber et al. argued that only peripheral blood cytokines were associated with SIRS and extracerebral organ dysfunction [162]. Evidence from an in vitro study indicated that inflammatory cytokines (i.e., TNF-α and IL-1β) can cause apoptosis of bovine brain endothelial cells [163]. The disrupted BBB aids in inflammatory cell migration, promoting intracerebral inflammation and secondary injuries [95,164]. These cytokines exert different properties based on the tissue and cell type. Some metabolites that have been consistently observed as the byproducts of these biochemical processes are collectively referred to as inflammation markers. In the next section, we discuss these extremely useful clinical tools for monitoring the rapid physiological changes among patients. The clinical associations of some of the most commonly measured cytokines (i.e., TNF-α and IL-6) and inflammation biomarkers (i.e., CRP and procalcitonin) are summarized in Table 1.

TNF-α is a proinflammatory cytokine that regulates cell growth, differentiation, and apoptosis [176]. It also mediates inflammatory diseases such as rheumatoid arthritis, systemic lupus erythematosus, and ankylosing spondylitis [177,178]. TNF-α is found to be elevated in the brain parenchyma, serum, and CSF 2–10 days after aSAH [156,179,180,181,182]. It causes neuron apoptosis [183] and BBB disruption [184] in in vivo brain injury models. Kimura et al. proposed that TNF-α dose-dependently promotes cerebral endothelial cell injury via the apoptotic caspase-3 pathway [185]. Matrix metalloproteinase-9 upregulation by TNF-α may also cause BBB damage [186]. In contrast, Ruigrok et al. found that lower serum TNF-α expression was associated with worse functional outcomes (Glasgow Outcome Scale (GOS) ≤ 3 at 3 months) in patients with aSAH [187].

IL-1β promotes inflammation, apoptosis, and immune response to tissue injury and infection [188,189,190]. IL-1β is produced by both innate and adaptive immune cells (i.e., macrophages, neutrophils, astrocytes, microglia, B lymphocytes, etc.) [191]. Intereukin-1Ra is secreted by the same cells that produce IL-1β and is the natural endogenous inhibitor of IL-1 cytokine [192]. IL-1Ra showed potential in treating rheumatoid arthritis and stroke [192,193]. In multivariate analysis, Bjerkne et al. found that the Glasgow Outcome Scale-Extended (GOSE) score of patients with SAH at one year follow-up was associated with their IL-1Ra serum levels during hospitalization [167]. They also found that IL-1β was elevated in CSF within 1 week [181] and that IL-1Ra was upregulated in systemic circulation on day 10 [167]. Korostynski et al. used whole-transcriptome sequencing to show that the IL-1 pathway was elevated in the peripheral mononuclear blood cells after aSAH [194]. Huang et al. proposed that IL-1 and ICAM-1 in peripheral blood and basilar artery might mediate cerebrovascular wall thickening, the vasospasm, and cortex neuron apoptosis via the P38-MAPK signal pathway, as discovered in a murine SAH model [195].

The IL-1 signal further promotes IL-6 production in macrophages, lymphocytes, fibroblasts, endothelial cells, glial cells, and neurons [196,197]. There is no evidence that the IL-6 gene polymorphism affects the risk or outcome of patients with aSAH [187,198]. Muroi et al. suggested that IL-6 upregulation in patients with aSAH follows a biphasic pattern, which represents early and late tissue injury [131]. IL-6 may help microglia migrate into the arterial wall and produce endothelin, which causes vasoconstriction [157]. Systemic IL-6 also increases liver CRP synthesis, an acute-phase protein in systemic inflammation that may further promote inflammation [199,200].

Transforming growth factor-β (TGF-β) is an anti-inflammatory cytokine produced by microglia and T regulatory cells [201] and was found to be elevated in the brain parenchyma, serum, and CSF following aSAH [202,203]. It was suggested that the anti-inflammatory properties of TGF-β might reduce neuronal injury and enhance clinical function among patients with aSAH [204,205]. However, evidence shows that TGF-β may also cause post-aSAH hydrocephalus via a fibrogenic effect [67,206]. Chen et al. suggested that post-aSAH hydrocephalus may be prevented through TGF-β inhibition, as indicated by the results from a mouse model using TGF-β-inhibitory-siRNA. The author suggested that reduced brain parenchymal TGF-β expression was associated with attenuated inflammatory reaction and improved neurological outcomes [207], contradicting previous studies. Further studies are required to determine the role of TGF-β in post-aSAH hydrocephalus and neuroinflammation.

## 8. Biomarkers of Inflammation: Acute-Phase Protein, MMP-9, and RNA Molecules

Acute-phase proteins (i.e., CRP, procalcitonin, complement proteins, haptoglobin, etc.) are powerful biomarkers of inflammatory response in infection, trauma, and autoimmune illnesses [208] induced by proinflammatory cytokines in systemic circulation. CRP is primarily produced by hepatocytes under stimulation of IL-6, which is elevated in post-aSAH patients’ venous blood and CSF samples [209,210]. CRP has been reported to be upregulated in systemic circulation around the week of admission [131,172,211]. Poor neurological results (GOS ≤ 3) are associated with a higher CRP level [211]. The peak CRP level was reported to predict neurological deficits, notably in the group that did not receive surgical clipping (adjusted OR: 1.27; 95% CI: 1.066–1.516) [211]. Güresir et al. found that CRP can predict DCI (OR 2.5, 95% CI 1.1–6.2, *p* = 0.049) and unfavorable clinical outcomes (mRS ≥ 3, OR 3.8; 95% CI: 1.9–7.2, *p* < 0.001) in patients with WFNS grades 1 and 2 [173]. Yang et al. found that patients with a serum hs-CRP level above 6.6 mg/L at admission have increased odds of experiencing acute kidney injury (AKI) within 2 weeks after aSAH (OR = 1.2; 95% CI: 1.1–1.3; *p* = 0.003) [212]. In a recent meta-analysis [213], Ma et al. found that elevated serum CRP was associated with increased odds of DCI (OR: 1.30; 95% CI: 1.10–1.54; *p* = 0.002). It was suggested that CRP may actively promote the inflammatory reaction, especially in the complement protein pathway [214]. However, the exact role and mechanism of CRP in post-aSAH inflammation requires further investigation.

Procalcitonin, the precursor molecule of calcitonin, is secreted by the thyroid, as well as adipose and brain tissue [215,216,217]. Procalcitonin was thought to be a specific biomarker for bacterial infection; however, subsequent studies found that it was upregulated in diverse inflammatory responses [218,219]. Elevated procalcitonin may represent neuronal injury in SAH (i.e., DCI) [175]. In a prospective trial of 132 patients with aSAH, Veldeman et al. found that within 2 days of admission, the procalcitonin serum level was associated with clinical outcome and DCI, regardless of infection state [175]. Güresir et al. found that patients with aSAH with less neurological deficit at presentation had a worse functional outcome after 6 months if their procalcitonin level was higher on the admission day [173]. However, the role of procalcitonin in neuronal damage remains unclear.

The complement protein system may also mediate post-SAH neuroinflammation [220]. Complement protein levels were found to be elevated in the CSF and peripheral blood and were correlated with the occurrence of vasospasm, DCI, and worse clinical outcome [15,48,221,222,223]. The lectin complement activation pathway was found to be activated within 24 h after SAH [224], as detected by elevated mannose-binding lectin (MBL), ficolin family, and collectin levels [225]. Cai et al. found that patients with aSAH with higher 6-month mortality had higher admission blood MBL levels [223]. Higher plasma ficolin-1 levels were associated with worse initial clinical state, vasospasm, and DCI [226]. Studies demonstrated that patients with worse mRS scores at 3-month follow-up had higher CSF ficolin-1 and MBL levels (+1.87 and +1.69 ng/mL, respectively) [227]. The membrane attack complex (MAC, C5-C9 protein) was proposed to be particularly important in the inflammatory reaction. MAC activation may cause BBB disruption, ipsilateral cerebral cortex albumin accumulation, and bilateral cerebral hemisphere brain edema [228]; MAC activation may also induce vasospasm by targeting vascular smooth muscle cells [229]. Van Dijk et al. demonstrated that C5 protein blockage might reduce microglial activation and neuronal apoptosis in C5a receptor knockout mouse and C5 antibody treatment aSAH models [230]. The researchers also found that the allele rs17611 C5 gene was associated with worse clinical results, regardless of the plasma C5 protein levels.

Haptoglobin functions as an anti-inflammatory acute-phase protein and hemoglobin scavenger [93,231,232]. It may reduce inflammation by increasing macrophage IL-10 production [233]. Kantor et al. found that patients with aSAH with the haptoglobin α2-α2 genotype had worse mRS at 3-month follow-up (OR: 4.138; 95% CI: 1.024–16.733, *p* = 0.0463) [234], possibly owing to reduced efficiency in hemoglobin removal [235]. In a meta-analysis, Gaastra et al. found that the haptoglobin α1-α2 heterozygous genotype was associated with secondary aSAH injuries but not long-term clinical outcomes [236]. In addition, a mouse aSAH model showed that the α1 homozygous haptoglobin genotype was associated with reduced subarachnoid macrophage and neutrophil infiltration [237]. Haptoglobin may be a potential approach to inhibit the hemoglobin-induced inflammatory response through direct intrathecal administration [238].

On the contrary, albumin is downregulated during systemic inflammation, making it a negative acute phase indicator. Patients with aSAH with entry serum albumin levels below 3.5 gm/dL had worse neurological outcomes and a higher 3-month mortality rate (27.6% vs. 16.4%, *p* = 0.04) than control patients [239]. Zhang et al. also found that the CRP/albumin ratio was an independent predictor of worse clinical outcome (GOS ≤ 3) at 3 months (OR: 17.072; 95% CI: 5.159–56.491; *p* < 0.001) [240].

Matrix metalloproteinase-9 (MMP-9) is a zinc-dependent endopeptidase that controls extracellular matrix homeostasis [241,242] and is regulated by proinflammatory cytokines, chemokines, hormones, and neurotransmitters [243]. MMP-9 is produced in various inflammatory cells, such as neutrophils, macrophages, microglia, and lymphocytes [69,244,245]. It may cause tissue damage [246] by upregulating chemokines (i.e., CCL2, CCL3, and CCL5 via the PI3K/p-AKT/NF-B pathway) and cytokines (i.e., TNF-α) [247], enhancing inflammatory cell migration [248]. Elevated peripheral MMP-9 levels are correlated with 30-day-survival [249], higher DCI rates [250], worse 3-month functional outcome (mRS ≥ 3) [69], and increased vasospasm risk [251]. Genetic evidence also shows that MMP-9 gene polymorphisms (SNP rs42512) are associated with aSAH survival [252]. MMP-9 may cause BBB breakdown [253,254] via degradation of the extracellular matrix (e.g., gelatin, elastin, and type IV collagen) [241]. This supports the potential approach to reduce BBB disruption, cerebral edema, and neurological dysfunction through MMP-9 inhibition [255].

Many non-coding RNAs involved in the epigenetic regulatory process may also be potential markers of systemic inflammation [256]. Distinct long non-coding RNA (lncRNA) [257] and circular RNA (circRNA) [258] expressions in the inflammatory pathways were observed in peripheral leukocytes following aSAH. MicroRNA (miRNA) upregulation (i.e., hsa-miR-195-5p, hsa-miR-486-3p, and hsa-miR-193b-3p) and downregulation (i.e., hsa-miR-369-3p, hsa-miR-136-3p, and hsa-miR-410-3p) patterns were observed in the plasma of patients with aSAH [259]. Cai et al. suggested that circARF3 inhibits miR-31-5p expression and, subsequently, the inflammatory MyD88/NF-κb pathway, which protects the BBB [260]. While it is not yet commonly available in clinical settings, this is a field of research with great potential, owing to rapidly development of sequencing technologies.

## 9. Conventional Anti-Inflammatory Approaches

Efforts have been made to explore different approaches to tackle systemic inflammation following aSAH. This section summarizes the most common conventional anti-inflammatory methods, and biologics will be discussed in the next section. Table 2 was constructed to provide an overview of the latest relevant clinical trials.

Non-steroidal anti-inflammatory drugs (NSAIDs), one of the most common anti-inflammatory treatments, block cyclooxygenase (COX) to reduce pain, inflammation, and fever. Celecoxib [261] and NS398 [262] are COX-2 inhibitors that effectively reduced vasospasm and neurological deficits in animal trials. Improved overall activity and less BBB disruption were observed among the celecoxib-treated aSAH mouse group [263]. Diclofenac also helped reduce inflammation and improve the clinical outcomes among patients with aSAH [264]; however, antipyretics may increase the risk of hemodynamic instability; therefore, more studies are needed to ensure patient safety [265].

Corticosteroids, another commonly used class of anti-inflammatory agents, may help in reducing vasospasm and IL-6 production [266]. However, no consensus exists on whether post-aSAH patients should receive steroids due to conflicting results [267]. Mohney et al. suggested that dexamethasone given 1 week after aSAH could prevent SIRS (OR: 0.49; 95% CI: 0.28–0.86) and improve outcomes (mRS < 3) without increasing the risk of nosocomial infection [268]. Miller et al. reported contrary results in a retrospective study of 206 patients with aSAH, focusing on extended steroid use [269]. Although Czorlich et al. similarly reported increased complications, the authors found that dexamethasone may promote favorable outcomes (GOS ≥ 4 at 3 months post discharge) among patients who underwent a microsurgical clipping procedure (OR: 0.193; 95% CI: 0.06–0.621; *p* = 0.006) [270].

Albumin supplement is commonly administered to patients with aSAH, even in the absence of a clear pharmacological indication [271]. Wang et al. proposed that albumin may bind hemoglobin metabolite, reducing inflammation and neuronal complications [272]. Xie et al. suggested that intravenous albumin may reduce brain parenchyma microglia activation and neutrophil infiltration [273]. In an endovascular perforation SAH mouse model, albumin therapy was associated with reduced BBB disruption and improved neurocognitive results [274]. The ALISAH pilot trial indicated that albumin may dose-dependently reduce cerebral vasospasm and ischemia [275]. However, no active clinical trial on albumin and aSAH was found by the end of the literature search.

The main non-pharmacological approaches are extracorporeal cooling approaches, including the use of specialized pads [276] and esophageal cooling devices [277]. While the results are promising, larger comparative clinical trials are needed. In May 2022, a multicenter randomized controlled trial, INTREPID (Impact of Fever Prevention in Brain Injured Patients) (NCT02996266), will investigate the benefits of induced normothermia in patients with febrile stroke using an extracorporeal cooling pad system (i.e., Arctic Sun Temperature Management System) [278]. Alternatively, cervical sympathetic block is a new method used to inhibit systemic inflammation [279]. Zhang et al. used ropivacaine to inhibit the stellate ganglion of patients with aSAH, which led to a reduction in proinflammatory cytokines (IL-6, IL-1β, and TNF-α), vascular activity markers (ET-1), and neuronal injury indicators (NSE, S100β) in venous blood [280]. Improved GOS scores (54% vs. 32.6%, *p* = 0.001) and reduced neurological deficits (hemiplegia, 20.0% vs. 34.6%, *p* = 0.023) were observed in the treatment arm. This novel method is being studied in several clinical trials, which may provide more insights (Table 2).

**Table 2:** Recent clinical trials investigating post-aSAH complication management.

Clinical Trials Identifier	Title	Intervention	Mechanism	Phase	Status
NCT04728438 [281]	Effect of Targeted Temperature Management on Cerebral Autoregulation in Patients With Neurocritical Diseases	Targeted temperature management (TTM)	Inhibit fever and prevent cerebral autoregulation dysfunction	NA	Not yet recruiting
NCT04566991 [282]	Deferoxamine In the Treatment of Aneurysmal Subarachnoid Hemorrhage (aSAH) (DISH)	Deferoxamine (IV) (32/mg/kg low dose vs. 48 mg/kg high dose) at 0, 24, and 48 h.	Inhibit iron-related free radical formation	Phase 2	Recruiting
NCT05259514 [283]	CytoSorb SAH Trial	Connected to a CytoSorb Adsorber for 48 h through a central venous line, with minimum blood flow of 200 mL/min.	Reduce IL-6 plasma level	NA	Terminated (safety)
NCT03249207 [284]	SC IL-1Ra in SAH–Phase III Trial (SCIL)	Subcutaneous IL-1Ra twice daily (SC) within 3 days for up to 3 weeks.	Inhibit interlekin-1 activity	Phase 3	Recruiting
NCT05282836 [285]	Early Hydrogen-Oxygen Gas Mixture Inhalation in Patients With Aneurysmal Subarachnoid Hemorrhage (HOMA)	66% H2-33% oxygen O_2_ at 3 L/min via nasal cannula using the Hydrogen/Oxygen Generator for 7 days in the ICU.	Prevent cerebral vasospasm and DCI	NA	Not yet recruiting
NCT04696523 [286]	Effect of Xenon on Brain Injury After Aneurysmal Subarachnoid Hemorrhage (Xe-SAH)	Xenon inhalation in air/oxygen with standard of care.	Anti-inflammatory action	Phase 2	Not yet recruiting
NCT04876638 [287]	Minocycline for Aneurysmal Subarachnoid Hemorrhage (MASH)	10 mg/kg minocycline up to 700 mg for 4 days	MMP-9 inhibitor	Phase 2	Active, not recruiting
NCT05103566 [288]	Safety, Feasibility, and Efficacy of Non-invasive Vagus Nerve Stimulation (nVNS) in the Treatment of Aneurysmal Subarachnoid Hemorrhage (STORM)	gammaCore with 2min stimulations twice/session to the cervical branch of the vagus nerve (nVNS) thrice daily.	Parasympathetic nerve stimulation	NA	Recruiting
NCT04691271 [289]	Stellate Ganglion Block and Cerebral Vasospasm (BLOCK-CVS)	Stellate ganglion block on the ipsilateral side of the lesion with 0.5% ropivacaine 8–10 mL local anesthesia.	Sympathetic nerve inhibition	NA	Recruiting
NCT04512859 [290]	Stellate Ganglion Block in Preventing Cerebral Vasospasm Secondary to Subarachnoid Hemorrhage	Stellate ganglion block using 0.25% ropivacaine 10 mL during aneurysmal coil embolism surgery.	Sympathetic nerve inhibition	NA	Not yet recruiting
NCT05230134 [291]	Cervical Sympathetic Block in Patients With Cerebral Vasospasm	Cervical sympathetic ganglion nerve block.	Sympathetic nerve inhibition	NA	Not yet recruiting
NCT04126408 [292]	Safety and Efficacy of Non-invasive Vagus Nerve Stimulation in the Treatment of Headache in Subarachnoid Hemorrhage (VANQUISH)	gammaCore two-two minute non-invasive stimulations of the cervical branch of the vagus nerve every 5 h.	Parasympathetic nerve stimulation	NA	Completed (20 June 2022, no published result)
NCT00585559 [293]	Acetaminophen in aSAH to Inhibit Lipid Peroxidation and Cerebral Vasospasm	Acetaminophen (1 g/6 h) and N-acetylcysteine (0.5 g/hour).	Inhibit the formation of ferryl-oxo radical of the heme and lipid peroxidation	Phase 3	Active, not recruiting
NCT05132920 [294]	Fight INflammation to Improve Outcome After Aneurysmal Subarachnoid HEmorRhage (FINISHER)	Dexamethasone(3 × 8 mg dexamethasone daily for days 1–7 and 1 × 8 mg dexamethasone daily for days 8–21).	Anti-inflammatory glucocorticoid effect	Phase 3	Recruiting

## 10. Application of Targeted Therapy in aSAH Inflammation

Cytokine-targeted therapy has shown favorable results that have already been incorporated into standard treatment protocols for various autoimmune and inflammatory diseases. TNF-α inhibition has shown improved outcomes in treating patients with rheumatoid arthritis, psoriasis, and inflammatory bowel disease [295,296]. Results from a rabbit aSAH model indicate that adalimumab, an anti-TNF-α monoclonal antibody, helps to inhibit cerebral vasospasm [179]. The results show that plasma TNF-α (23.23 ± 1.29 vs. 44.86 ± 2.58 pg/mL, *p* < 0.05) and IL-1β (50.05 ± 2.21 vs. 55.00 ± 3.92 pg/mL) levels decreased significantly, as did brain parenchyma MMP-9 expression (0.85 ± 0.09 vs. 1.29 ± 0.20 ng/mg protein, *p* < 0.05). Anti-TNF-α antibodies also inhibit the neuronal apoptosis signaling pathway (i.e., proapoptotic protein BAX, phosphorylated-Erk), as observed in the neuronal tissue of a rat aSAH model [297]. Zhang et al. suggested that etanercept (biologic TNF inhibitor) indirectly inhibits the JNK pathway, which drives the inflammatory and apoptotic reactions in aSAH [298].

Similar to TNF-α, interleukins have been targeted in acute (i.e., COVID-19) and chronic (i.e., Crohn’s disease, SLE) inflammatory diseases [299,300]. Anakinra is one of the most widely used IL-1-competitive inhibitors. In a phase II randomized control trial involving 136 patients with aSAH, subcutaneous anakinra injection lowered blood inflammatory markers (i.e., IL-6 and CRP) and improved functional outcomes (i.e., GOSE score at 6 months) [301]. A phase III trial is planned to further examine the effects of subcutaneous anakinra on clinical outcomes (NCT03249207). Tocilizumab is another popular IL-6 pathway drug used to treat severe COVID-19 [302]. In a rabbit SAH model, tocilizumab was shown to prevent vasospasm, reduce neuronal apoptosis, prevent blood clots, and reduce plasma IL-6 levels [303].

A recently completed randomized controlled phase II clinical study (CLASH–Complement C5 Antibodies for decreasing brain injury after aSAH) examined the neuroinflammation-reducing effects of intravenous eculizumab, a complement C5 monoclonal antibody. However, no published result was available at the end of the literature search.

An interesting approach is to target epigenetic regulation using non-coding RNA molecules. MALAT1, a long non-coding RNA, was identified as a prognostic marker of non-small cell lung cancer [304]. Zhou et al. proposed that si-MALAT1, the MALAT1-targeting siRNA, may improve neurological outcomes in patients with aSAH [305]. si-MALAT1 may disrupt the interaction between MALAT1 and miR-499-5p, which inhibits SRY-box transcription factor 6 (SOX6), a crucial transcription factor that causes neuronal apoptosis and ROS production. In addition, specific miRNAs may reduce neuronal injury and cerebral vasospasm (e.g., miR-146a inhibits the TLR4 signaling pathway [306] and miR-195-5p inhibits NF-κb expression). Intravenous miR-193b-3p inhibits the HDAC3/NF-κb pathway and improves neurological results [259]. Deng et al. upregulated anti-inflammatory HMOX1 protein by inhibiting miR-24 expression, subsequently maintaining BBB integrity and reducing systemic inflammatory cytokines [307]. Chen et al. also found that reduced miR-502-5p expression inhibits the NF-κb/p65 inflammatory pathway and promotes neuron survival [308].

## 11. Conclusions

The systemic inflammatory state following SAH is the consequence of complex interactions between the nervous and immune systems, which have profound effects on all organ systems. Previous studies have demonstrated that the inflammatory responses mediate various post-aSAH complications and are correlated with the patient outcomes. Therefore, it is important to detect and evaluate the extent of systemic inflammation in patients with aSAH. The chemical signals involved in the communication between the nervous and immune system may be useful biomarkers to monitor inflammatory responses and potential therapeutic targets. Previous research has shed light on the advantages of the various treatment approaches; however, further research is warranted to gain a deeper understanding of the pathophysiological mechanisms of systemic inflammation following aSAH and their clinical implementation.

## Figures and Tables

**Table 1:** Common cytokines and biomarkers for the prediction of aSAH complications and clinical outcomes.

Cytokine/ Biomarker	Increase (↑)/Decrease (↓)	Clinical Outcome	Secondary Injury	Human/Animal	Author (Year)	Comment
Serum TNF-α	↑ Day 2–3	Poor outcome (mRS ≥ 3) 3 months (*p* = 0.0004) 6 months (*p* = 0.017)	Vasospasm (*p* > 0.05)	Human (*n* = 52)	Chou et al. (2012) [165]	
	↑ Day 0–12	Poor outcome (WFNS categories IV and V) (OR: 2.0; 95% CI: 0.4–9.0)	DCI (hazard ratio 0.6, 95% CI 0.1–2.4)	Human (*n* = 67)	Beeftink et al. (2011) [166]	
	↑ Day 0	Poor outcome (GOSE 1–4) 1 year (*p* = 0.02)	DCI (*p* > 0.05)	Human (*n* = 58)	Bjerkne Wenneberg et al. (2021) [167]	
Serum IL-6	↑ Day 2–3	Poor outcome (mRS ≥ 3) 3 months (*p* > 0.05) 6 months (*p* = 0.05)	Vasospasm (*p* > 0.05)	Human (*n* = 52)	Chou et al. (2012) [165]	
	↑ Day 0	Poor outcome (GOSE 1–4) 1 year (*p* = 0.01)	DCI (*p* > 0.05)	Human (*n* = 58)	Bjerkne Wenneberg et al. (2021) [167]	
	↑ Day 0	Poor outcome (mRS ≥ 3) 6 months (*p* = 0.018)	n/a	Human (*n* = 81)	Höllig, et al. (2015) [168]	
	↑ Day 3	Poor outcome (mRS ≥ 3) 6 months (*p* = 0.012)	DIND (*p* > 0.05)	Human (*n* = 43)	Al-Tamimi et al. (2019) [158]	
	↑ Day 4	Poor outcome (GOS 1–3) 1 year (*p* = 0.315)	Vasospasm (*p* = 0.912)	Human (*n* =44)	Vlachogiannis et al. (2019) [169]	
	↑Day 3, 7	Poor outcome (GOS 1–3) 3 months (*p* < 0.05)	DIND (*p* < 0.05)	Human (*n* = 138)	Muroi et al. (2013) [131]	Biphasic upregulation pattern
	↑ Day 2	Poor Outcome (mRS ≥ 4 at discharge) (*p* < 0.01)	EBI (*p* = 0.03)	Human (*n* = 71)	Savarraj J et al. (2018) [70]	
	n/a	n/a	Final rate of change * correlated to DCI (*p* = 0.03)	Human (*n* = 179)	McMahon CJ et al. (2013) [170]	* Means daily increment over the last two readings
	↑ Day 1–13	Poor outcomes (GOS 1–3) (mRS ≥ 3) Timing not specified (*p* < 0.05)	DIND, casospasm, chronic hydrocephalus (*p* < 0.05)	Human (*n* = 80)	Chaudhry et al. (2017) [171]	
CRP [96]	↑ Day 0	Poor outcomes (GOSE 1–4) 1 year (*p* = 0.01)	DCI (*p* > 0.05)	Human (*n* = 58)	Bjerkne Wenneberg et al. (2021) [167]	
	↑ Day 0	Poor outcomes (mRS ≥ 3) 6 months (*p* = 0.163)	n/a	Human (*n* = 81)	Höllig et al. (2015) [168]	
	↑ > day 7	Poor outcomes (GOS 1–3) 3 months (*p* < 0.05)	DIND (*p* < 0.05)	Human (*n* = 138)	Muroi et al. (2013) [131]	
	↑ Day 0	Poor outcome (GOS 1–3) 3 months (*p* = 0.004)	DCI (*p* < 0.001)	Human (*n* = 178)	Juvela et al. (2012) [172]	
	↑ Day 0	Poor outcome (mRS 3–6) 6 months (*p* = 0.001; OR: 3.2; 95% CI: 1.6–6.6)	n/a	Human (*n* = 231)	Güresir et al. [173]	
	↑ Day 0, 7	n/a	DIND (*p* < 0.05)	Human (*n* = 46)	Kubo et al. (2008) [174]	
Procalcitonin	↑ Day 0	Poor outcome (mRS 3–6) 6 months (*p* = 0.004; OR: 26.0; 95% CI: 2.9–235.5)	DCI (AUC 0.752, 95% CI 0.735–0.886)	Human (*n* = 231)	Güresir et al. [173]	
	↑ Day 0–2	Poor outcome (GOSE 1–4) 1 year (*p* = 0.025)	DCI (*p* = 0.025)	Human (*n* = 132)	Veldeman et al. (2020) [175]	

Abbreviation: TNF-α: tumor necrosis factor-α; IL-6: interleukin 6; CRP: C-reactive protein; IL-1Ra: interleukin-1 receptor antagonist; mRS: modified Rankin scale; WFNS: World Federation of Neurosurgical Societies; GOSE: Glasgow Outcome Scale—Extended; GOS: Glasgow Outcome Scale; DCI: delayed cerebral ischemia; DIND: delayed ischemic neurological deficit.

## Data Availability

Not applicable.
